# Detection of Sleep Apnea from Single-Lead ECG Signal Using a Time Window Artificial Neural Network

**DOI:** 10.1155/2019/9768072

**Published:** 2019-12-23

**Authors:** Tao Wang, Changhua Lu, Guohao Shen

**Affiliations:** ^1^School of Computer and Information, Hefei University of Technology, Hefei, Anhui, China; ^2^School of Software, Hefei University of Technology, Hefei, Anhui, China

## Abstract

Sleep apnea (SA) is a ubiquitous sleep-related respiratory disease. It can occur hundreds of times at night, and its long-term occurrences can lead to some serious cardiovascular and neurological diseases. Polysomnography (PSG) is a commonly used diagnostic device for SA. But it requires suspected patients to sleep in the lab for one to two nights and records about 16 signals through expert monitoring. The complex processes hinder the widespread implementation of PSG in public health applications. Recently, some researchers have proposed using a single-lead ECG signal for SA detection. These methods are based on the hypothesis that the SA relies only on the current ECG signal segment. However, SA has time dependence; that is, the SA of the ECG segment at the previous moment has an impact on the current SA diagnosis. In this study, we develop a time window artificial neural network that can take advantage of the time dependence between ECG signal segments and does not require any prior assumptions about the distribution of training data. By verifying on a real ECG signal dataset, the performance of our method has been significantly improved compared to traditional non-time window machine learning methods as well as previous works.

## 1. Introduction

Sleep apnea (SA) is a ubiquitous sleep-related respiratory disease [1] that can be roughly divided into central sleep apnea (CSA) caused by lack of input of the central nervous system, obstructive sleep apnea (OSA) caused by collapse of the upper respiratory tract, and hypopnea caused by airflow reduction [2]. It can occur even hundreds of times at night, and its long-term occurrences can lead to some serious cardiovascular and neurological diseases such as acute coronary syndrome, congestive heart failure, and excessive sleep during the day. Studies have shown that approximately 5% of women and 14% of men in the United States have SA syndrome [3], and the incidence of this disease has increased in various populations worldwide. Clinically, if the patient's AHI exceeds five times an hour, it is usually diagnosed with sleep apnea syndrome and must be treated [2].

Polysomnography (PSG) is a commonly used diagnostic method for SA [3]. It requires suspected patients to sleep in the lab for one to two nights and records about 16 signals including airflow signal, respiratory effort, electroencephalogram (EEG), electrocardiogram (ECG), and oxygen saturation (SaO_2_) [3, 4]. Patients need to wear a large number of wires and electrodes, and there should be an expert to monitor. These shortcomings hinder the widespread implementation of PSG in public health applications. Therefore, an alternative technique is needed that can be comfortably applied to SA detection. To solve this problem, several researchers have proposed using a single-lead signal (i.e., pulse oximetry, snoring, electroencephalograph, or electrocardiogram) to automatically diagnose SA [5–7]. Among these options, the single-lead ECG is the most concerned signal because a single-lead ECG signal can be used to derive the respiratory signal (EDR), and the combination of the respiratory signal and the RR intervals of the ECG signal can effectively detect sleep apnea [8, 9]. Besides, it is easy to record using a wearable device.

Therefore, in recent years, researchers have proposed some automatic sleep apnea detection methods based on a signal-lead ECG signal. For example, Khandoker et al. develop a SA patient identification method based on support vector machine (SVM) and features extracted from ECG-derived respiratory (EDR) signals and RR intervals. In their work, more than 90% of subjects in the test set are correctly classified [10]. Varon et al. employ two novel features extracted from ECG signals and ECG-derived respiratory signals, and the time-domain features obtained from RR intervals to diagnose SA [11]. The sensitivity and accuracy of each segment are 84.7% and 84.7%, respectively. Shouldice et al. propose a classification method for detecting SA in pediatric patients by integrating a 1-minute ECG signal into the quadratic discriminant analysis (QDA), thereby achieving an accuracy of 84% on a per-recording basis and 72.1% on a per-segment basis [9]. Sharma and Sharma develop an LS-SVM SA detection method using Hermite basic function feature obtained from RR intervals; the sensitivity and accuracy of their work are 79.5% and 83.8%, respectively [12].

Existing studies mainly extract various features from the current ECG signal segment and then use the traditional machine learning method to directly construct the SA detection model. These methods imply the assumption that SA relies only on the current ECG signal segment. However, studies have shown that SA has time dependence [3, 13]; that is, the SA of the ECG segment at the previous moment has an impact on the current SA diagnosis. For example, Song et al. propose a Hidden Markov model (HMM) using the temporal dependence existing among segments [3]. The sensitivity and accuracy are 82.6% and 86.2%, respectively. Meanwhile, Li et al. followed Song et al. work and develop a SA detection method based on the Hidden Markov model and deep neural network (DNN) [13]. The sensitivity and accuracy of their work are 88.9% and 84.7%, respectively. However, these methods ignore the fact that HMM needs training data to satisfy a specific distribution [14, 15], resulting in limited performance improvements. In this study, we propose a time window artificial neural network method for SA detection. Compared with existing methods, our method not only takes advantage of the time dependence between ECG signal segments but also does not require any prior assumptions about the distribution of training data [16, 17]. By verifying on a real ECG signal dataset, the performance of our method has been significantly improved compared to traditional non-time window machine learning methods as well as previous works. To our knowledge, our study is the first study to use a time window for sleep apnea detection.

## 2. Materials and Methods

### 2.1. Dataset

In this study, the PhysioNet Apnea-ECG dataset provided by Dr. Thomas Penzel of Phillips University is used to build and test our proposed method [18, 19]. This dataset consists of a released set and a withheld set, each set containing 35 ECG signal recordings extracted from PSG recordings with a 16-bit resolution, a sampling rate of 100 Hz. For each 1-minute segment of these ECG signal recordings, an expert combines other signals (i.e., respiration, oxygen saturation) in PSG to annotate SA or normal. The total is 34,313 1-minute ECG segments, of which the released set contains 17,045 and the withheld set has 17,268. There is no difference between hypopneas and apneas in the annotation. Furthermore, these ECG signal recordings are divided into three classes (A, B, and C) according to the AHI value (number hypopnea of and apnea events per hour). When the AHI value of the recording is less than 5, it is defined as class C, that is, normal. When the recording AHI value is greater than 10, it is defined as class A, that is, apnea. Between the two recordings, the recording with an AHI of 5 or more during sleep is defined as class B (borderline apnea). The details of the dataset are listed in [Supplementary-material supplementary-material-1]. Generally, the released set is used for model building and parameter estimation, and the withheld set is used to test the model.

### 2.2. Preprocessing

**Figure 1:**
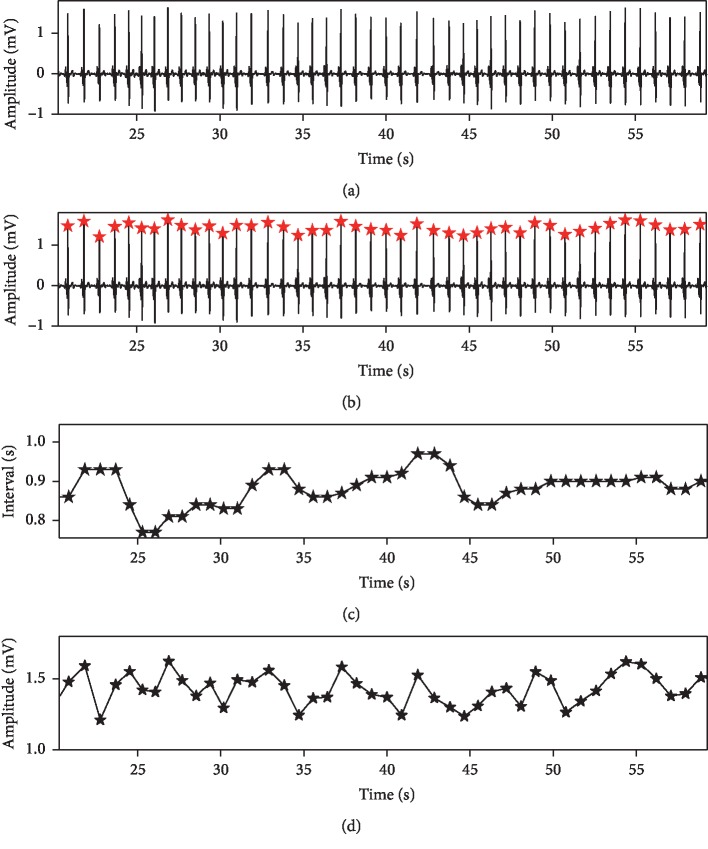
An illustration of the RR intervals and R-peak amplitudes extracted from 1-minute ECG segment (6,000 sampling points). (a) Original ECG signal. (b) Located R peak using the Hamilton algorithm. (c) Extracted RR intervals. (d) Extracted amplitudes.

Previous studies have shown that the RR intervals and R-peak amplitudes of ECG signal contain critical information about the occurrence of SA [3, 10, 11, 13]. Hence, a preprocessing procedure is applied to obtain the RR intervals and R-peak amplitudes from the ECG signal. Notably, the original ECG signal contains a lot of noise (i.e., baseline drift and power frequency noise), and we have used the FIR bandpass filter with passband 3∼45 Hz to denoise the original ECG signal before processing. The RR interval is defined as the time interval between two consecutive R peaks [3]. We use the Hamilton algorithm [20] to locate the R peaks and then use the located R peaks to calculate the RR intervals. Since there are physiologically uninterpretable points in the generated RR intervals, we follow Chen et al. [21] using a median filter to eliminate those underlying abnormal values. The R-peak amplitudes represent the value of R peaks of the ECG signal, which reflects respiratory activity, and we extract its value directly. It is worth noting that the signal constructed by the R-peak amplitudes is also called the EDR signal [22].  Figure 1 shows an illustration of the RR intervals and R-peak amplitudes extracted from the original ECG signal.

### 2.3. Feature Extraction

Various features are extracted from RR intervals and R-peak amplitudes in previous studies. Here, we combine 12 features extracted from RR intervals and 6 features obtained from R-peak amplitudes to construct our model. Below, we describe these features in detail.

#### 2.3.1. RR Intervals Features

The features consist of 6 time-domain features and 6 frequency-domain features. The 6 time-domain features are as follows: MRR (mean of RR intervals), MHR (mean of heart rates), RMSSD (root mean square of differences between adjacent RR intervals), SDNN (standard deviation of RR intervals), NN50 (number of adjacent RR intervals exceeding 50 milliseconds), and pNN50 (NN50 divided by the number of RR intervals) [23, 24].

(1) $$\begin{array}{c} \text{MRR}=\tilde{\text{RR}}=\frac{\sum_{n=1}^{N}\text{RR}\left(n\right)}{N}, \end{array}$$

(2) $$\begin{array}{c} \text{RMSSD}=\sqrt{\frac{\sum_{n=2}^{N}{\left(\text{RR}\left(n\right)-\text{RR}\left(n-1\right)\right)}^{2}}{N-1}}, \end{array}$$

(3) $$\begin{array}{c} \text{SDNN}=\sqrt{\frac{\sum_{n=1}^{N}{\left(\text{RR}\left(n\right)-\tilde{\text{RR}}\right)}^{2}}{N-1},} \end{array}$$

where RR(*n*) represents the value of *n*′th RR interval, and *N* is the number of RR intervals.

For the frequency features, we first calculate the corresponding power spectral density using the Welch method [25, 26] with FFT (*s*, *N* = 256) and then extract 6 features using the power of each frequency component including VLF (very low frequency, 0∼0.04 Hz), LF (low frequency, 0.04∼0.15 Hz), HF (high frequency, 0.15∼0.4 Hz), LF/HF (ratio of LF to HF), LF/(LF + HF) (ratio of LF to LF and HF), and HF/(LF + HF) (ratio of HF to LF and HF). It is worth noting that since the amount of original samples in each window is small, by following [26, 27], we interpolate the original samples.

#### 2.3.2. R-Peak Amplitudes Features

Studies show that the power spectral density of the R-peak amplitudes and RR intervals has similar characteristics that can be used for SA diagnosis [22, 28]. Therefore, in addition to the frequency features of RR intervals, we also extract the above 6 frequency features (VLF, LF, HF, LF/(LF + HF), and HF/(LF + HF)) from the R-peak amplitudes for building the model.

Since the distribution of these features is very different, after feature extraction, we use the following formula to normalize the features:

(4) $$\begin{array}{c} x^{*}=\frac{x-\tilde{x}}{\sigma }, \end{array}$$

where $\tilde{x}$ represents the mean of the feature and *σ* is the standard deviation of the feature.

### 2.4. Artificial Neural Network

#### 2.4.1. Multilayer Perceptron (MLP)

**Figure 2:**
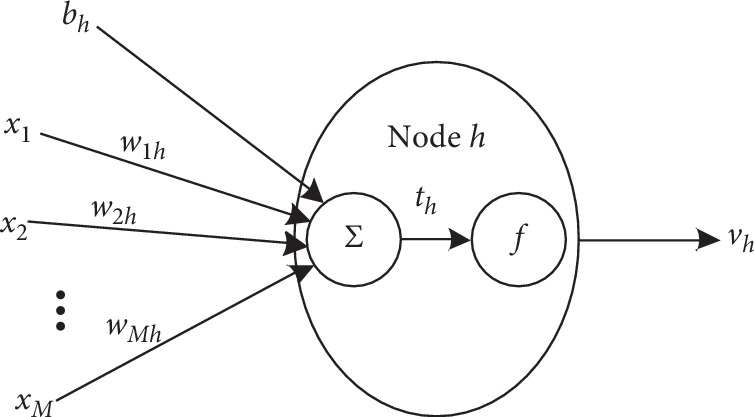
The basic structure of neuron.

Multilayer perceptron (MLP) is an artificial neural network that models complex functions [29]. It consists of three or more layers (an input layer, one or more hidden layers, and an output layer) of nodes. Compared with traditional methods (i.e., HMM), MLP does not require any prior assumptions regarding the distribution of training data [16, 17], avoiding the influence of data distribution on performance. Meanwhile, it can achieve better performance with simple setting [16]. Therefore, we use MLP as a benchmark method in this study. For the structure of the network, Hornik et al. [30] shows that 1 hidden layer backpropagation network with sufficient hidden nodes can implement a universal approximator. So, we use 1 hidden layer MLP for sleep apnea detection. Each node of the hidden layer is connected with the input layer by weights and the output is the sum of the inputs under the activation function, defined as follows:

(5) $$\begin{array}{c} v_{h}=f\left(\overset{M}{\underset{m=1}{\sum }}\omega_{mh}^{\left(1\right)}x_{m}+b_{h}^{\left(1\right)}\right), \end{array}$$

where *b*_*h*_^(1)^ is the bias, *ω*_*mh*_^(1)^ is the connection weight between the input *x*_*m*_ and the neuron *h*, *M* is the number of features, and *f* is the activation function. The common activation functions are sigmoid, tanh, relu, and maxout [31]. Here, we use the relu activation function because it is easier to optimize. *v*_*h*_ is the output of the neuron.  Figure 2 shows the basic structure of a neuron. Similarly, the output layer nodes are given by the following equation:

(6) $$\begin{array}{c} y_{c}=\overset{H}{\underset{h=1}{\sum }}\omega_{hc}^{\left(2\right)}v_{h}+b_{c}^{\left(2\right)}, \end{array}$$

where *ω*_*hc*_^(2)^ are the weights connecting the output with the hidden nodes, *b*_*c*_^(2)^ are the bias connecting the hidden with the output node, and *H* is the number of the hidden layer nodes. In this study, we use the Kolmogorov superposition theorem [31, 32] to set *H* to 2*∗M*+1. In MLP, the weights and biases are optimized by using the BP algorithm to minimize the sum of mean squared error (MSE) *E* between the target *t*_*c*_ and realistic output *y*_*c*_.

#### 2.4.2. Combining with Time Window

**Figure 3:**
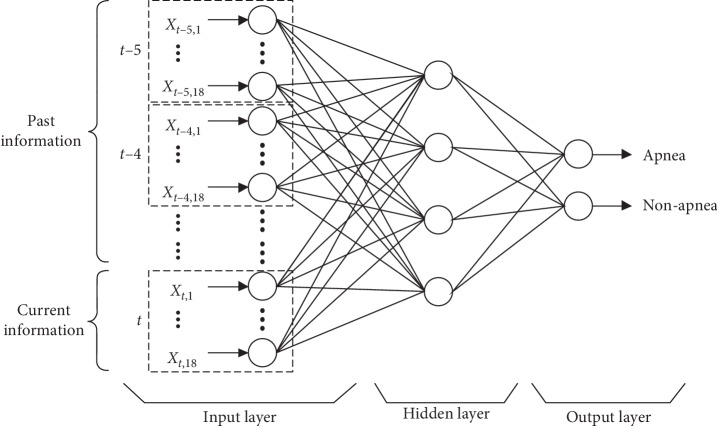
Time window MLP algorithm scheme. The upper part of MLP input represents past information and the lower part of MLP input represents current information. In this study, the time window is 5, and the hidden layer of our final model has 181 nodes based on the Kolmogorov superposition theorem.

Studies have shown that there is timing dependence between ECG signal segments. However, previous studies use features selected from the current ECG signal segment as input. This usually means that the output from the algorithm depends only on the current ECG signal segment features. Therefore, to consider the prior input features, we propose a time window based artificial neural network method. For a specific time window size, the method collects all past features within the time window and current features as input to the learning algorithm. The time window used in this study is 5 ([Supplementary-material supplementary-material-1]), which is selected by cross validation in the released set [33].  Figure 3 illustrates the scheme of our proposed method. It is notable that the time window used in this study is a moving time window in which each subsequent window differs by only one data point.

### 2.5. Performance Evaluation

The performance is evaluated by accuracy, specificity, sensitivity, and area under the curve (AUC).

(7) $$\begin{array}{c} \text{Accuracy}=\frac{\text{TP}+\text{TN}}{\text{TP}+\text{TN}+\text{FP}+\text{FN}}, \end{array}$$

(8) $$\begin{array}{c} \text{Specificity}=\frac{\text{TN}}{\text{TN}+\text{FP}}, \end{array}$$

(9) $$\begin{array}{c} \text{Sensitivity}=\frac{\text{TP}}{\text{TP}+\text{FN}}, \end{array}$$

where TP and TN refer to the number of true samples classified as positive and the number of true samples classified as negative, respectively. FP and FN refer to the number of false samples classified as positive and the number of false samples classified as negative, respectively.

## 3. Results

In this study, a time window with a neural network (TW-MLP) is proposed for SA detection. To verify the effectiveness of this method, in the following parts, we will compare it with traditional non-time window machine learning methods in per-segment SA detection and per-recording classification.

### 3.1. Per-Segment SA Detection

As mentioned above, each ECG signal of these recordings is segmented into a 1-minute segment, and the per-segment SA detection refers to determining whether each 1-minute segment is a SA or normal. It is an important basis for diagnosing SA for suspicious patients. Therefore, we first analyze the performance of our proposed method as well as several non-time window methods including linear discriminant analysis (LDA), support vector machine (SVM), logistic regression (LR), and MLP in per-segment SA detection.  Table 1 lists the accuracy, sensitivity, specificity, and AUC of these methods. As shown in Table 1, the traditional non-time window methods have similar performance; the accuracy is around 81.0%, and the AUC is between 0.866 and 0.885. Compared with the traditional non-time window machine learning methods, the performance of our proposed method TW-MLP is significantly improved. For example, compared with the best accuracy traditional non-time machine learning window LDA method, the accuracy, sensitivity, specificity, and AUC of TW-MLP are improved by 5.5%, 14.2%, 0.3%, and 0.065, respectively. Similarly, MLP, after adopting the time window, the accuracy, sensitivity, specificity, and AUC are increased by 5.9%, 10.8%, 3%, and 0.06, respectively. In general, our proposed method has the best performance compared to traditional non-time window machine learning methods, and the time window can effectively improve the per-segment SA detection performance.

### 3.2. Per-Recording Classification

**Figure 4:**
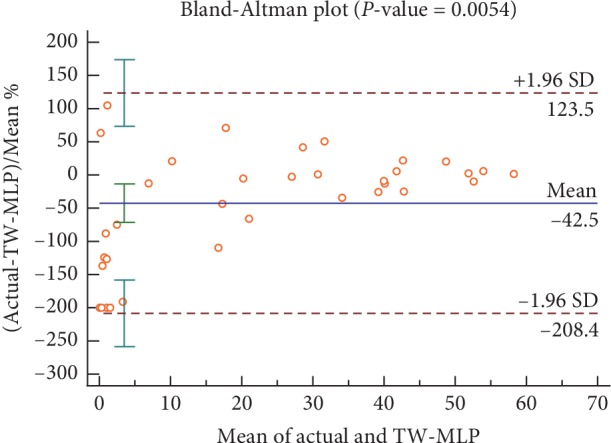
The Bland–Altman plot of TW-MLP AHI and actual AHI.

After obtaining the per-segment SA detection results, we further classify the recording based on the above results, that is, to diagnose whether the patient has SA symptoms. It is worth noting that normal people also have short-term SA phenomena. Clinically, the AHI value is used for diagnosis. If the AHI value for the recording is greater than 5, the patient is considered to have sleep apnea disorder. Otherwise, it is diagnosed as normal. The AHI value refers to the number of minutes with apnea per hour, expressed as follows:

(10) $$\begin{array}{c} \text{AHI}=\frac{60}{T}*\text{num of SA segments,} \end{array}$$

where *T* denotes the minute of the recording.  Table 2 shows the comparison results of TW-MLP and traditional non-time window machine learning methods. Due to the small test recordings (35 recordings), we, following [3, 12], use the correlation value between the experimentally determined AHI and the actual AHI (Corr.) to further ensure the reliability of the results. It can be noticed from Table 2 that, compared with traditional non-time window machine learning methods, the performance of TW-MLP has improved significantly. For example, the accuracy, sensitivity, specificity, AUC, and correlation value of LR are 91.4%, 100.0%, 75.0%, 1.000%, and 0.850, respectively. While the corresponding values of TW-MLP are 97.1%, 100.0%, 91.7%, 1.000, and 0.935, respectively. Besides, to ensure the reliability of clinical applications, we also use the Bland-Altman plot to verify the consistency of TW-MLP AHI and actual AHI, as shown in Figure 4. The results show that our method has a good consistency. In general, in per-recording classification, the time window can effectively improve the performance of our method, and the result is consistent with the actual AHI.

## 4. Discussion

In the previous section, we have verified the effectivity of TW-MLP. In this section, we will discuss the effect of time window size and compare it with previous works.

### 4.1. Effect of Time Window Size

**Figure 5:**
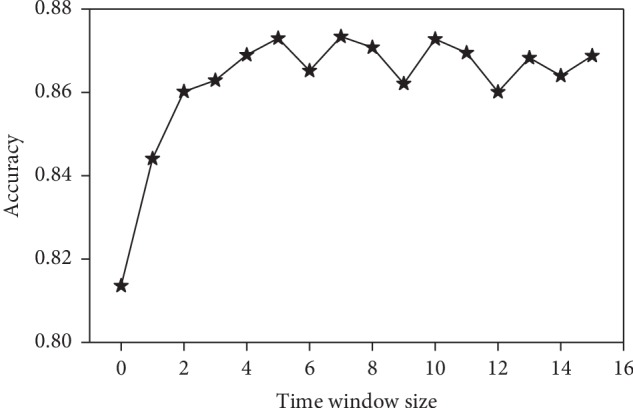
The accuracy of TW-MLP in different time windows. The time window size equal to 0 represents the non-time window artificial neural network (MLP).

In this study, we develop a SA detection method using a time window artificial neural network. It is natural to consider the effect of time window size on performance. Therefore, here we analyze the effect of time window size, as shown in Figure 5. It should be pointed out that the nodes in the hidden layer are recalculated according to the Kolmogorov superposition theorem as the window size changes, and the time window size equal to 0 represents the non-time window artificial neural network (MLP). It can be seen from Figure 5 that the accuracy increases significantly with the increase of the time window at the beginning. When the highest peak is reached, the accuracy decreases slowly. The best accuracy (87.34%) is obtained when the time window size is equal to 7 (the time window size used in this study is different from this value, which is selected by cross validation ([Supplementary-material supplementary-material-1])). These results show that the time window can effectively improve the performance, and the performance will be significantly improved with the increase of the time window size within a certain range. However, we should avoid overfitting due to excessive time window. Normally, cross validation is a good way to choose the size of the time window.

### 4.2. Compared with Previous Works

At present, there are some SA detection works based on the PhysioNet Apnea-ECG database. To further verify the effectiveness of our proposed method, we compare it with these works. It is worth noting that due to the different data preprocessing used in these works, the samples are slightly different, and a direct comparison between these methods is not available [13].  Table 3 lists the performance of our work and related works in per-segment SA detection. As can be seen from Table 3, our work achieves the best performance. For example, the accuracy, sensitivity, and specificity of Song et al. [3] are 86.2%, 82.6%, and 88.4%, respectively, while our work gets an accuracy of 87.3%, sensitivity of 85.1%, and specificity of 88.7%. In addition, the per-recording classification of related works is shown in Table 4. As shown in Table 4, compared with most previous works, our work has better or comparable performance. For example, our work has the same accuracy as Sharma and Sharma [12] in per-recording classification, but our work has a higher correlation value. It should be pointed that in Li et al. [13], to get the best performance, they directly use the withheld set to search for the optimal parameters, which leads to overfitting of the results.

### 4.3. Robustness Evaluation

**Figure 6:**
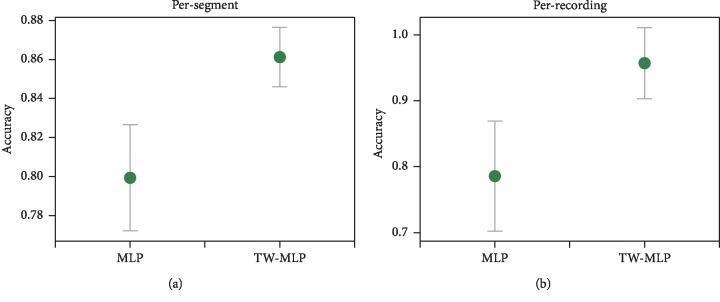
Cross-validation results for MLP and TW-MLP on the (a) per-segment SA detection and (b) per-recording classification. The green dot represents the average accuracy of the cross validation, while the gray line refers to the corresponding 95% confidence interval (CI).

The PhysioNet Apnea-ECG dataset used in this paper is a relatively small dataset. Using a single withheld dataset to validate our method may result in biased results. In order to solve this problem, here we use the 7-fold cross validation to test the robustness of our method under different datasets. The entire dataset (70 recordings) is divided into 7 parts, each time using 6 parts for training, and the rest for testing.  Figure 6 shows the average accuracy and 95% CI of the cross validation of the per-segment SA detection and per-recording classification before (MLP) and after (TW-MLP) using the time window. As can be seen from Figure 6, our method TW-MLP has a consistent performance on the different datasets, and its performance on the per-segment SA detection and per-recording classification is significantly improved compared to the non-time window method. For example, MLP obtains an accuracy of 79.9% ± 2.7% on the per-segment SA detection, while TW-MLP achieves an accuracy of 86.1% ± 1.5%, and the average accuracy is improved by 6.2%.

## 5. Conclusion

The goal of this study is to develop a fast and portable automatic sleep apnea detection method. In order to achieve this goal, based on the time dependence found by Song et al. [3], we propose a time window artificial neural network (TW-MLP) method using a single-lead ECG signal. Compared with existing works, our method not only takes advantage of the time dependence between ECG signal segments but also avoids the impact of data distribution on performance. By validating in the PhysioNet Apnea-ECG dataset, our method can effectively detect sleep apnea and further improve its performance compared to traditional non-time window machine learning methods as well as previous works. Meanwhile, the Bland-Altman plot of TW-MLP AHI and actual AHI show that our method is consistent and can be used as an alternative to the physician for initial screening. Although we achieve good performance, there are still some limitations and possible improvements. The dataset used in this study does not separately annotate different types of apnea, and there is no difference between hypopnea and apnea [18]. In future research, we will consider combining multiple databases to detect different types of apnea. Furthermore, we extract various useful features with reference to previous studies and achieve better performance. However, the feature extraction process is susceptible to the experience of the researcher. In recent years, several studies have shown that the convolutional neural network (CNN) can automatically extract good features [36], which can be considered as feature extraction steps.

## Figures and Tables

**Table 1:** Performance of TW-MLP and traditional non-time window machine learning methods on per-segment SA detection.

Method	Accuracy (%)	Sensitivity (%)	Specificity (%)	AUC
LR	81.5	72.0	87.4	0.880
LDA	81.8	70.9	88.4	0.880
SVM	80.6	72.1	85.8	0.866
MLP	81.4	74.3	85.7	0.885
TW-MLP	87.3	85.1	88.7	0.945

**Table 2:** Performance of TW-MLP and traditional non-time window machine learning methods on per-recording classification.

Method	Accuracy (%)	Sensitivity (%)	Specificity (%)	AUC	Corr.
LR	91.4	100.0	75.0	1.000	0.850
LDA	88.6	100.0	66.7	1.000	0.855
SVM	82.9	100.0	50.0	0.975	0.875
MLP	82.9	100.0	50.0	0.986	0.851
TW-MLP	97.1	100.0	91.7	1.000	0.935

**Table 3:** Comparison with previous works in per-segment SA detection.

Reference	Classifier	Accuracy (%)	Sensitivity (%)	Specificity (%)
Song et al. [3]	HMM-SVM	86.2	82.6	88.4
Varon et al. [11]	LS-SVM	84.7	84.7	84.7
Li et al. [13]	Decision fusion	83.8	88.9	88.4
Sharma and Sharma [12]	LS-SVM	83.4	79.5	88.4
Our work	TW-MLP	87.3	85.1	88.7

**Table 4:** Comparison with previous works in per-recording classification.

Reference	Classifier	Accuracy (%)	Sensitivity (%)	Specificity (%)	Corr.
Sharma and Sharma [12]	LS-SVM	97.1	95.8	100.0	0.841
Álvarez et al. [34]	LR	89.7	92.0	85.4	—
Morillo and Gross [35]	PNN	93.8	92.4	95.9	—
Li et al. [13]	Decision fusion	100.0	100.0	100.0	—
Song et al. [3]	HMM-SVM	97.1	95.8	100.0	0.860
Our work	TW-MLP	97.1	100.0	91.7	0.935

## Data Availability

The data and code used to support the findings of this study are included in Supplementary Information Files.

## Supplementary Materials

Supplementary Materials Table S1: the details of the PhysioNet Apnea-ECG dataset. Figure S1: the 5-fold cross-validation accuracy for different time windows on the released set.
