# Supplementary material for: Detection of Sleep Apnea from Single-Lead ECG Signal Using a Time Window Artificial Neural Network
Source: Biomed Res Int. 2019 Dec 23;2019:9768072. doi: 10.1155/2019/9768072 (PMC6948296; doi:10.1155/2019/9768072)
Supplement: Supplementary Materials — Table S1: the details of the PhysioNet Apnea-ECG dataset. Figure S1: the 5-fold cross-validation accuracy for different time windows on the released set. [file 9768072.f1.docx]

**Table S1.** The details of the PhysioNet Apnea-ECG dataset ^a^.

| Record | Length | non-apnea | apnea | hours | AI^b^ | HI^c^ | AHI^d^ | Age | Sex | height | weight |
| --- | --- | --- | --- | --- | --- | --- | --- | --- | --- | --- | --- |
| a01 | 490 | 20 | 470 | 9 | 12.5 | 57.1 | 69.6 | 51 | M | 175 | 102 |
| a02 | 529 | 109 | 420 | 9 | 57.2 | 12.3 | 69.5 | 38 | M | 180 | 120 |
| a03 | 520 | 274 | 246 | 9 | 38.4 | 0.7 | 39.1 | 54 | M | 168 | 80 |
| a04 | 493 | 40 | 453 | 9 | 73.4 | 4 | 77.4 | 52 | M | 173 | 121 |
| a05 | 455 | 179 | 276 | 8 | 35 | 6 | 41 | 58 | M | 176 | 78 |
| a06 | 511 | 305 | 206 | 8 | 16.6 | 8.1 | 24.7 | 63 | M | 179 | 104 |
| a07 | 512 | 190 | 322 | 9 | 46 | 17 | 63 | 44 | M | 177 | 105 |
| a08 | 502 | 313 | 189 | 7 | 32 | 10 | 42 | 51 | M | 179 | 88 |
| a09 | 496 | 115 | 381 | 9 | 23.1 | 8.6 | 31.7 | 52 | M | 178 | 82 |
| a10 | 518 | 418 | 100 | 6 | 11 | 10 | 21 | 58 | M | 176 | 78 |
| a11 | 467 | 245 | 222 | 8 | 11 | 3 | 14 | 58 | M | 168 | 103 |
| a12 | 578 | 44 | 534 | 10 | 70 | 10.2 | 80.2 | 52 | M | 173 | 121 |
| a13 | 496 | 252 | 244 | 9 | 32 | 10 | 42 | 51 | M | 179 | 88 |
| a14 | 510 | 127 | 383 | 8 | 17.3 | 37.4 | 54.7 | 51 | M | 175 | 102 |
| a15 | 511 | 143 | 368 | 9 | 46 | 6 | 52 | 60 | M | 176 | 113 |
| a16 | 483 | 163 | 320 | 7 | 17 | 24 | 41 | 44 | M | 177 | 105 |
| a17 | 486 | 328 | 158 | 5 | 21 | 12 | 33 | 40 | M | 179 | 96 |
| a18 | 490 | 52 | 438 | 9 | 75.5 | 6.9 | 82.4 | 52 | M | 178 | 82 |
| a19 | 503 | 298 | 205 | 9 | 34 | 0 | 34 | 55 | M | 178 | 90 |
| a20 | 511 | 196 | 315 | 9 | 35 | 6 | 41 | 58 | M | 176 | 78 |
| b01 | 488 | 469 | 19 | 2 | 0.12 | 0.12 | 0.24 | 44 | F | 170 | 63 |
| b02 | 518 | 425 | 93 | 5 | 14 | 5 | 19 | 53 | M | 176 | 85 |
| b03 | 442 | 369 | 73 | 4 | 22 | 2 | 24 | 53 | M | 176 | 85 |
| b04 | 430 | 420 | 10 | 1 | 0.7 | 0 | 0.7 | 42 | M | 180 | 64 |
| b05 | 434 | 377 | 57 | 3 | 2 | 3 | 5 | 52 | M | 180 | 135 |
| c01 | 485 | 485 | 0 | 0 | 0 | 0 | 0 | 31 | M | 184 | 74 |
| c02 | 503 | 502 | 1 | 0 | 0 | 0 | 0 | 37 | M | 180 | 83 |
| c03 | 455 | 455 | 0 | 0 | 0 | 0 | 0 | 39 | M | 184 | 65 |
| c04 | 483 | 483 | 0 | 0 | 0 | 0 | 0 | 41 | F | 180 | 65 |
| c05 | 467 | 464 | 3 | 0 | 0 | 0 | 0 | 28 | F | 169 | 57 |
| c06 | 469 | 468 | 1 | 0 | 0 | 0.25 | 0.25 | 28 | F | 171 | 65 |
| c07 | 454 | 450 | 4 | 0 | 0 | 0 | 0 | 30 | F | 168 | 56 |
| c08 | 535 | 535 | 0 | 0 | 0 | 0 | 0 | 42 | M | 180 | 64 |
| c09 | 469 | 467 | 2 | 0 | 0 | 0 | 0 | 37 | M | 180 | 83 |
| c10 | 432 | 431 | 1 | 0 | 0 | 0 | 0 | 27 | M | 184 | 72 |
| x01 | 524 | 149 | 375 | 9 | 46 | 17 | 63 | 44 | M | 177 | 105 |
| x02 | 470 | 261 | 209 | 7 | 27.3 | 10.4 | 37.7 | 46 | M | 167 | 69 |
| x03 | 466 | 454 | 12 | 1 | 0.13 | 0 | 0.13 | 44 | F | 170 | 63 |
| x04 | 483 | 483 | 0 | 0 | 0 | 0 | 0 | 39 | M | 184 | 65 |
| x05 | 506 | 190 | 316 | 9 | 34 | 0 | 34 | 55 | M | 178 | 90 |
| x06 | 451 | 451 | 0 | 0 | 0 | 0 | 0 | 31 | M | 170 | 66 |
| x07 | 510 | 270 | 240 | 8 | 11 | 10 | 21 | 58 | M | 176 | 78 |
| x08 | 518 | 194 | 324 | 8 | 48 | 0 | 48 | 55 | M | 178 | 90 |
| x09 | 509 | 342 | 167 | 5 | 1.8 | 16.7 | 18.5 | 43 | M | 177 | 80 |
| x10 | 511 | 415 | 96 | 6 | 3 | 7 | 10 | 39 | M | 170 | 131 |
| x11 | 458 | 445 | 13 | 1 | 2 | 3 | 5 | 52 | M | 180 | 135 |
| x12 | 528 | 471 | 57 | 4 | 21 | 12 | 33 | 40 | M | 179 | 96 |
| x13 | 507 | 215 | 292 | 8 | 18.5 | 0.2 | 18.7 | 57 | M | 171 | 97 |
| x14 | 491 | 52 | 439 | 9 | 61.2 | 18.3 | 79.5 | 38 | M | 180 | 120 |
| x15 | 499 | 299 | 200 | 8 | 11.3 | 4.6 | 15.9 | 63 | M | 179 | 104 |
| x16 | 516 | 451 | 65 | 4 | 22 | 2 | 24 | 53 | M | 176 | 85 |
| x17 | 401 | 400 | 1 | 0 | 0 | 0 | 0 | 27 | F | 158 | 53 |
| x18 | 460 | 458 | 2 | 0 | 0 | 0 | 0 | 27 | M | 184 | 72 |
| x19 | 488 | 81 | 407 | 9 | 50.1 | 6.1 | 56.2 | 54 | M | 168 | 80 |
| x20 | 514 | 250 | 264 | 8 | 34 | 9 | 43 | 51 | M | 179 | 88 |
| x21 | 511 | 391 | 120 | 4 | 14 | 5 | 19 | 53 | M | 176 | 85 |
| x22 | 483 | 481 | 2 | 0 | 0 | 0 | 0 | 27 | F | 158 | 53 |
| x23 | 528 | 409 | 119 | 3 | 9.9 | 4.4 | 14.3 | 43 | M | 177 | 80 |
| x24 | 430 | 429 | 1 | 0 | 0 | 0 | 0 | 31 | M | 170 | 66 |
| x25 | 511 | 220 | 291 | 9 | 48 | 0 | 48 | 55 | M | 178 | 90 |
| x26 | 521 | 177 | 344 | 9 | 14.4 | 0.7 | 15.1 | 57 | M | 171 | 97 |
| x27 | 499 | 11 | 488 | 9 | 71 | 4 | 75 | 60 | M | 176 | 113 |
| x28 | 496 | 62 | 434 | 9 | 71 | 4 | 75 | 60 | M | 176 | 113 |
| x29 | 471 | 471 | 0 | 0 | 0 | 0 | 0 | 41 | F | 180 | 65 |
| x30 | 512 | 186 | 326 | 9 | 17 | 24 | 41 | 44 | M | 177 | 105 |
| x31 | 558 | 42 | 516 | 10 | 86.8 | 6.7 | 93.5 | 29 | F | 183 | 100 |
| x32 | 539 | 114 | 425 | 9 | 63.3 | 8.5 | 71.8 | 29 | F | 183 | 100 |
| x33 | 474 | 471 | 3 | 0 | 0.13 | 0 | 0.13 | 28 | F | 169 | 57 |
| x34 | 476 | 472 | 4 | 0 | 0.38 | 0 | 0.38 | 30 | F | 168 | 56 |
| x35 | 484 | 484 | 0 | 0 | 0 | 0 | 0 | 31 | M | 184 | 74 |

^a^ The table is originally provided by Dr. Thomas Penzel.

^b^ The apnea index (AI) is the number of apneas observed per hour.

^c^ The HI is the number of hypopneas observed per hour.

^d^ The apnea-hypopnea index (AHI) is defined as the sum of AI and HI.


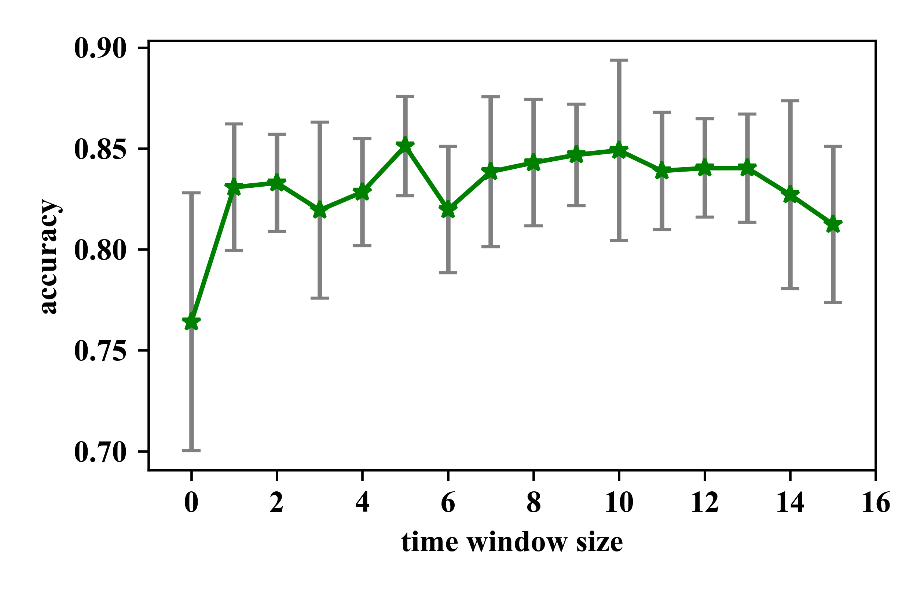


**Figure S1.** The 5-fold cross-validation accuracy for different time windows on the released set. The green line represents the average 5-fold cross-validation accuracy for different time windows, while the gray line represents 95% CI for 5-fold cross-validation. It should be noted that if the dataset used in the time window selection contains the withheld set, the final model will overfit. The time window used in this study is 5, because the average accuracy is the highest, and 95% CI is small.
